# Chemical ubiquitination for decrypting a cellular code

**DOI:** 10.1042/BJ20151195

**Published:** 2016-05-11

**Authors:** Mathew Stanley, Satpal Virdee

**Affiliations:** * MRC Protein Phosphorylation and Ubiquitylation Unit, University of Dundee, Dundee DD1 5EH, Scotland, U.K.

**Keywords:** atypical, chemical biology, chemoenzymatic, isopeptide, ligation, post-translational modification, semisynthesis, ubiquitin, ubiquitination, ubiquitylation

## Abstract

The modification of proteins with ubiquitin (Ub) is an important regulator of eukaryotic biology and deleterious perturbation of this process is widely linked to the onset of various diseases. The regulatory capacity of the Ub signal is high and, in part, arises from the capability of Ub to be enzymatically polymerised to form polyubiquitin (polyUb) chains of eight different linkage types. These distinct polyUb topologies can then be site-specifically conjugated to substrate proteins to elicit a number of cellular outcomes. Therefore, to further elucidate the biological significance of substrate ubiquitination, methodologies that allow the production of defined polyUb species, and substrate proteins that are site-specifically modified with them, are essential to progress our understanding. Many chemically inspired methods have recently emerged which fulfil many of the criteria necessary for achieving deeper insight into Ub biology. With a view to providing immediate impact in traditional biology research labs, the aim of this review is to provide an overview of the techniques that are available for preparing Ub conjugates and polyUb chains with focus on approaches that use recombinant protein building blocks. These approaches either produce a native isopeptide, or analogue thereof, that can be hydrolysable or non-hydrolysable by deubiquitinases. The most significant biological insights that have already been garnered using such approaches will also be summarized.

## INTRODUCTION

Modification of substrate proteins with ubiquitin (Ub) and polyubiquitin (polyUb) chains of diverse topology is now firmly established as a crucial regulator of eukaryotic biology [1–3], but the study of such conjugates remains particularly challenging. The process of ubiquitination occurs via the concerted action of a series of enzymes (E1s, E2s and E3s) which results in the transfer of Ub to the Nε amino group of lysine residues within substrate proteins, or, to Ub itself in the case of chain formation [1]. Ub has seven lysine residues (K6, K11, K27, K29, K33, K48 and K63) and together with the Nα amino group of the initiating methionine residue (Met1), gives rise to eight potential polyUb signals [3]. Proteomic efforts over the past decade have revealed that all eight linkage types exist in cells and their abundance can be quantified [4–8]. Ub can also form branched, heterotypic, chains that contain more than one linkage type [9–11]. Ub can also form heterologous chains with ubiquitin-like proteins (Ubls) such as small ubiquitin-like modifier (SUMO) and neural precursor cell expressed, developmentally down-regulated 8 (NEDD8) [12–15]. Furthermore, thousands of ubiquitinated substrates have been identified that can be modified at multiple sites. It has therefore emerged that the versatile topology of polyUb and its attachment to distinct positions within substrate proteins forms the basis of an expanding code that regulates a wide array of cellular processes [3,16,17].

The first evidence polyUb chains were significant was provided in 1985 when it was shown that polyUb attachment to substrate proteins accelerates substrate degradation by the proteasome [18]. Four years later it became apparent that the type of Ub linkage is not selected arbitrarily and exquisite selectivity towards a distinct lysine residue can be achieved [19]. An enzymatic system was identified and cloned shortly thereafter that produced K48 chains [20]. Fortuitously, the system was amenable to *in vitro* reconstitution and provided a scalable platform for preparing K48-linked Ub chains. A scalable enzymatic platform was subsequently developed for the production of K63-linked Ub chains [21].

As a feature of these systems was the ability to prepare large quantities of K48 and K63 linkages as free, unanchored chains, this greatly facilitated biochemical study and accelerated our understanding of the cellular N-terminal intein fragment roles of these linkage types. It followed that to begin to fully appreciate the cellular significance of substrate ubiquitination, general methods that allow the production of substrate proteins that are site-specifically modified with Ub species of defined topology are needed. However, platforms for preparing the remaining ‘atypical’ linkages were less obliging and our knowledge of E3 substrates remains poor to this day [22,23]. Increasing evidence in support of the cellular importance of these linkages combined with unbiased proteomic studies revealing ubiquitination sites on thousands of proteins [4–8,24], placed strong incentive to develop enzyme-independent methods for protein ubiquitination. Such chemical methods would be expected to have long-term utility as when the number of lysine acceptor sites throughout the proteome is considered, systematic identification of a compatible enzymatic system to site-specifically, and efficiently, ubiquitinate substrates of interest with defined topology is a formidable challenge. Fortunately, the chemical biology community have provided a battery of powerful methodologies over the past decade that has begun to address this challenge and these have been extensively reviewed in the chemical biology literature [25–29].

**Figure 1:**
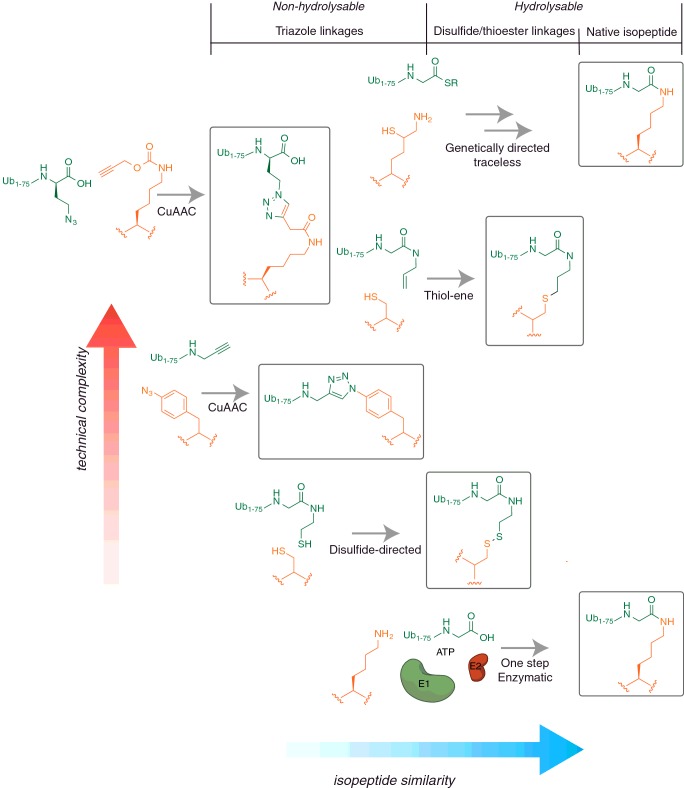
A structural comparison of non-native linkages compared with the native isopeptide bond present in ubiquitinated proteins Linkages fall into three main chemical classes: native isopeptide linkages, disulfide/thioether linkages and triazole linkages, which are further subdivided based on their ability to be proteolytically cleaved by DUBs (hydrolysable or non-hydrolysable). The relative position of a particular linkage type illustrates its structural similarity to the native isopeptide bond (blue axis), versus the technical complexity associated with its generation (red axis). Chemical methods that furnish a native isopeptide bond tend to be the most technically challenging.

The aim of this review is to provide an overview of the techniques that are available for preparing Ub conjugates and Ub chains with particular focus on approaches that use recombinant protein building blocks rather than those that are reliant on synthetic peptide synthesis. Arguably, these approaches are more general and are easier to implement in typical biology research labs and therefore have the potential to provide immediate impact to many Ub researchers. These approaches either produce a native isopeptide, or analogue thereof, that can be hydrolysable or non-hydrolysable (Figure 1 and Table 1). The most significant biological insights that have already been garnered using such approaches will also be summarized.

**Table 1:**
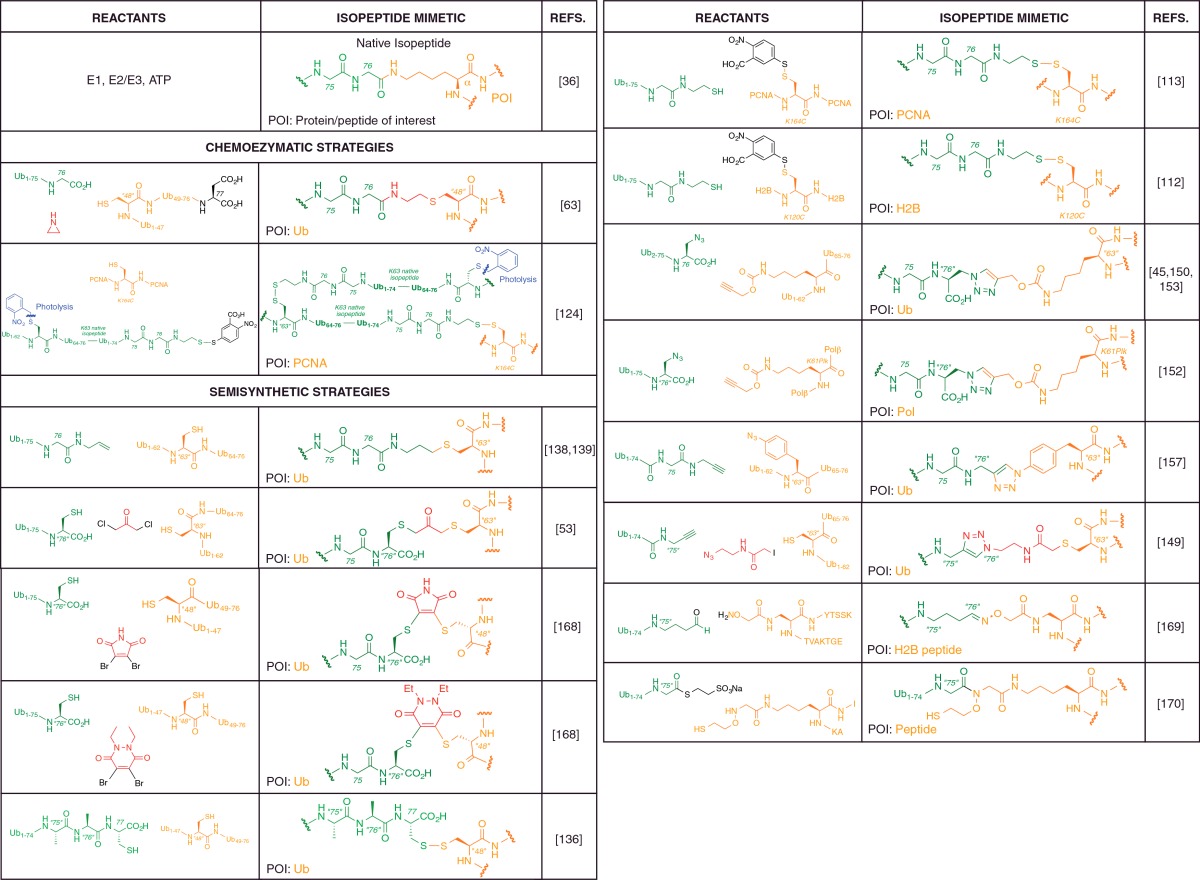
Literature examples of non-native isopeptide linkages incorporated into Ub chains or ubiquitylated proteins and peptides *in lieu* of the native isopeptide linkage Reference to the native isopeptide bond structure allows comparison of the non-native linkages with the atomic connectivity of the native isopeptide linkage. The table colour coding is given as follows: orange, protein/peptide of interest (POI) present in a reactant or in the non-native linkage; green, Ub (or polyUb) present in a reactant or in the non-native linkage (independent of the POI); red, small molecule reactant which forms a component of the non-native linkage; black, chemical auxiliary present in a reactant but not in the final conjugate conjugate; blue, blocking functionality that permits iterative chain assembly. The main reagents for a specific ligation procedure are provided but not all small molecule reagents necessary have been specified. Atom labels, X, denotes the native amino acid position whereas “X”, denotes a non-native amino acid or mutation introduced at position X (for example, 76 and ‘76’, referring to Gly-76 of Ub). Mutations of the native Ub amino acid sequence at positions not directly relevant to the isopeptide bond are given in parentheses within the reactant chemical structures.

Over the past 5 years numerous enzymatic systems have been described that have, in part, addressed the historical absence of methods for the enzymatic assembly of atypical Ub chains [30–35] (for a recent review see [36]). However, the enzymatic assembly reactions described only produce seven of the eight possible linkage types, and for the production of native K6, K11, K29 and K33 chains, two-step assembly is usually required (i.e. E2/E3 assembly followed by deubiquitinating enzyme ‘editing’ [37]). Furthermore, a significant task that cannot be carried out enzymatically in a general manner is the creation of complex Ub topologies such as defined heterotypic and heterologous linkages [22]. Many of the technologies developed thus far can be used in a modular fashion thereby harnessing the convenience of enzymatic methods with the generality and site-specificity of chemical methods, which should prove to be particularly powerful for studying the effects of substrate ubiquitination.

## CUTTING OUT THE MIDDLE MAN

**Figure 2:**
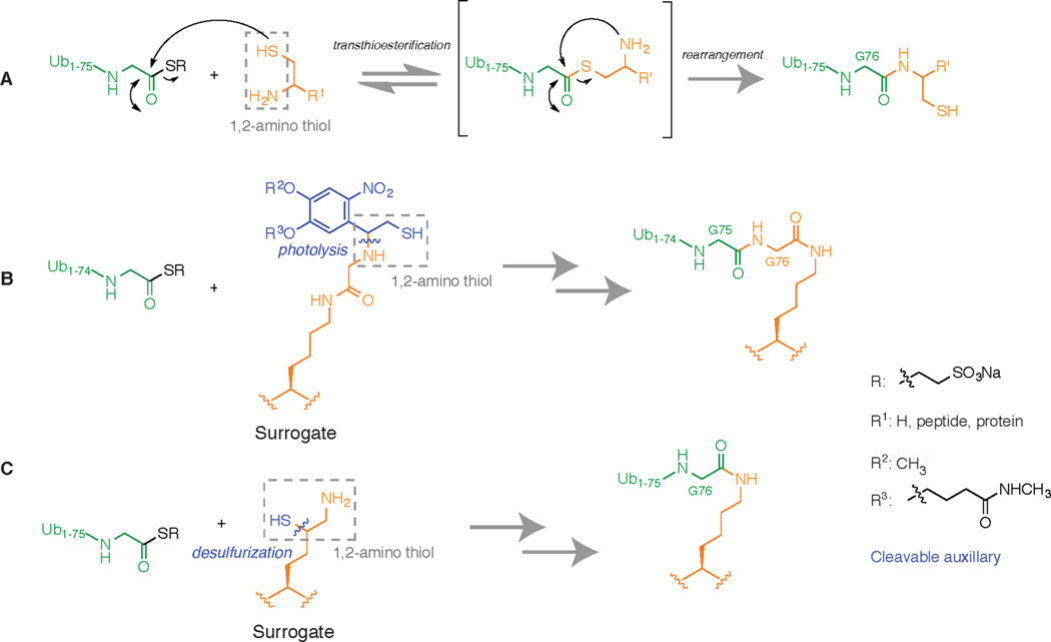
Chemoselective chemistry between protein thioesters and 1,2-amino thiols (**A**) A protein thioester can undergo chemoselective amide bond formation with species bearing 1,2-amino thiol functionality. In the simplest embodiment, the 1,2-amino thiol can be cysteamine (R^1^=H), providing a means to linearly append a thiol group to the C-terminus of a protein. (**B**) A lysine surrogate prepared by preformation of an isopeptide bond between the Nε amino group of lysine and glycine, and introduction of a fragmentable 1,2-amino thiol moiety on to the glycine N-terminus. The surrogate can undergo EPL with Ub1-75-SR followed by photolytic fragmentation to form a native isopeptide bond. (**C**) Introduction of a 1,2-amino thiol moiety by appending a thiol group to the δ-C atom of lysine. EPL followed by desulfurization forms a native isopeptide bond.

The challenges associated with making defined Ub conjugates without enzymes are classic chemical problems which have been largely solved a myriad ways for small molecule synthesis [38]. The first being, how can one selectively modify a particular occurrence of a chemical group (amino groups) in the presence of multiple instances of the same chemical group? The second problem being, how can the poorly reactive carboxylate group, present at the C-terminus of Ub, be selectively activated or replaced, to drive a conjugation reaction? The first point can be addressed by incorporating a lysine surrogate amino acid that confers inherent chemoselectivity. This can be achieved by the inclusion of a removable auxiliary appendage on the surrogate, which directs site-specific formation of a native isopeptide linkage when mixed with Ub appropriately activated at its C-terminus (Figure 2).

In some instances C-terminal activation has been solved for us by Nature as the Ub E1 activating enzyme (UBA1) selectively activates the C-terminus of Ub by formation of a labile thioester in an ATP-dependent manner [39,40]. Importantly, this process can be exploited for the large-scale chemoenzymatic synthesis of Ub activated at its C-terminus as a small molecule thioester that can readily undergo chemoselective chemistry [41,42]. Furthermore, UBA1 can selectively activate the C-terminus of native and non-hydrolysable polyUb chains allowing, in principle, the selective functionalization of polyUb species [41,43–45]. Small molecule protein thioesters of recombinant origin provide the basis of the extremely powerful semisynthetic strategy known as expressed protein ligation (EPL) [46]. EPL relies on the recombinant expression of proteins of interest as C-terminal fusions with engineered inteins which by virtue of their intrinsic splicing activity, provides a general route to the preparation of C-terminal protein thioesters [47]. As selective activation of the C-terminus provides a powerful means of selective protein functionalization, intein technology has become the mainstay of many semisynthetic methodologies [48,49].

EPL is an extension of the native chemical ligation (NCL) reaction which uses entirely synthetic peptide thioesters that can undergo chemoselective peptide bond formation with synthetic peptides bearing N-terminal cysteines [50]. The characteristic feature of cysteine that ensures chemoselectivity towards thioesters is the presence of a 1,2-amino thiol moiety (Figure 2A). However, the 1,2-amino thiol can be derived from reaction components other than N-terminal Cys containing peptides which allows the development of strategies for isopeptide bond formation that will be described later (Figures 2B and 2C).

However, the primary challenge with enzyme-independent ubiquitination is associated with the incorporation of the unnatural surrogate amino acid into the protein of interest. Chemical peptide synthesis grants the ability to incorporate, in principle, any chemical functionality into a peptide but routine synthesis is limited to ∼50 amino acids [51]. An optimized protocol for the total chemical synthesis of Ub has been developed that enables its unrestricted manipulation but this requires specialist chemical methodology [42]. Challenges arise with protein substrates because if a large protein is to be modified, the synthetic peptide needs to be inserted into the remaining protein components and then folded. Due to a restricted repertoire of unnatural functionality that can be incorporated into recombinant protein at the genetic level, surrogate amino acids that achieve chemoselectivity but furnish non-native linkages have been very popular. Moreover, these approaches produce important reagents in their own right as the non-natural linkage is typically non-hydrolysable by the enzymes that reverse Ub conjugation, deubiquitinating enzymes (DUBs) [52] (Figure 1 and Table 1). This enables distinct experiments to be carried out that could not be achieved with native conjugates due to enzymatic cleavage of the isopeptide linkage [45,53].

## TRACELESS UBIQUITINATION BY SEMISYNTHESIS

### The semisynthesis of K120 ubiquitinated H2B

Histone 2B (H2B) is ubiquitinated at K120 (uH2B) in cells and this post-translational modification had been associated with the regulation of gene expression and with increased levels of histone 3 (H3) methylation at position 79 (H3K79) [54–56]. Methylation was known to be carried out by the methyltransferase Dot1L but whether uH2B stimulated this activity directly or whether accessory factors were involved remained unknown [57–59]. Seminal work from the Muir lab demonstrated the enzyme-independent formation of a native isopeptide between Ub and a synthetic peptide corresponding to the C-terminal H2B tail that harboured the K120 ubiquitination site. This was achieved using the EPL methodology that hitherto had only been used for the *linear* semisynthesis of proteins [46,48]. Chemical synthesis of the H2B peptide enabled the incorporation of an unnatural lysine surrogate at position K120 with a fragmentable 1,2-amino thiol moiety attached to the lysine side chain enabling formation of the branched isopeptide [60]. The unnatural amino acid consisted of lysine with a glycine residue pre-isopeptide linked to the Nε amino group which served as a surrogate for Gly-76 of Ub (Figure 2B). The C-terminus of Ub was then selectively activated as a thioester using intein technology. To ensure production of an entirely native conjugate, C-terminal thioesterification of Ub missing Gly-76 was required (Ub1-75-SR; R is typically **−**CH_2_CH_2_SO_3_H). EPL was carried out between Ub1-75-SR and the synthetic peptide thereby forming an isopeptide bond. Elegantly, the auxiliary could be photolytically removed providing a mild and completely traceless route to ubiquitinated peptides.

Subsequently, it was demonstrated that the synthetic peptide could be appended to the complementary N-terminal H2B peptide by extended semisynthetic methods [61]. This allowed the traceless preparation of full-length uH2B in sufficient quantities for biochemical analysis. Incorporation of uH2B into reconstituted nucleosomes allowed unequivocal experiments to be carried out revealing that ubiquitination of H2B at K120 mediated direct cross-talk within a nucleosome by directing methylation of H3K79 by Dot1L [61]. As established protocols for nucleosome formation begin with denatured histones [62], and Ub is readily refoldable [63], the synthetic nature of the H2B polypeptide was of no consequence. Asymmetric dinucleosomes were also prepared to determine whether internucleosomal methylation could occur in *trans*. It was found that asymmetric nucleosomes were not methylated, suggesting that nucleosomes *in vivo* that are methylated at H3K79, but do not carry the Ub modification, were at one time ubiquitinated and subsequently subjected to DUB activity.

Limitations with this approach are that multiple ligation steps are required which is exacerbated with large protein targets and when the conjugation sites are not close to the substrate termini. There is also restricted generality because the design of the conjugate assembly tends to be tailored to a particular substrate. Furthermore, labs that do not have expertise with synthetic peptide synthesis might find it challenging to source the specialist peptide building blocks on the large scales required for protein semisynthesis. To alleviate the latter point, simplified approaches were developed, albeit with the introduction of C-terminal G76A mutation in Ub, that were reapplied to H2B and extended to histone 2A (H2A) [64,65]. In these contexts the mutation was shown to be functionally silent. Extending the utility of these simplified routes, DNA-barcoded nucleosome libraries (DNL) were constructed which contained distinct combinations of histone PTMs (acetylation, methylation and ubiquitination) [66]. Using semi-synthetically prepared PTM carrying histones, the authors were able to rapidly assemble a collection of 54 histone PTM modifications upon chemically defined nucleosomes. This library was then used for carrying out an ultrasensitive and rapid ChIP-seq based analysis in a platform suitable for profiling the binding preferences of various nuclear factors towards the varied histone PTM patterns displayed. Results obtained in many cases largely recapitulated known literature interactions and as such acted as a successful proof-of-principle application for the method.

## GENETIC METHODS FOR NATIVE UBIQUITINATION

### Genetically directed EPL for isopeptide bond formation

Two almost parallel communications subsequently reported an alternative lysine surrogate that could undergo EPL to form a native isopeptide bond [67,68]. In these embodiments, the 1,2-amino thiol moiety was installed on the lysine side chain by simply appending a thiol group at the δ (δ-thiolysine) or γ C-atom (a 1,3-amino thiol that can still undergo NCL [67]) of the lysine side chain (Figure 2C). Post ligation with full-length Ub thioester (Ub1-76-SR), the minimal thiol auxiliary could be removed by mild free-radical desulfurization [69], again yielding an entirely native isopeptide linkage. However, incorporation of the unnatural lysine surrogate was still limited to synthetic peptides.

To unlock the potential of this streamlined chemistry to mediate ubiquitination of recombinant proteins, efforts were undertaken to effect the genetic incorporation of δ-thiolysine by amber codon suppression using an evolved *Methanosarcina barkeri* pyrrolysyl-tRNA synthetase/tRNA_CUA_ (*Mb*PylRS/tRNA_CUA_) pair [70]. Genetic code expansion using wild type and evolved *Mb*PylRS/tRNA_CUA_ pairs had already been successfully employed to incorporate a range of unnatural lysine derivatives into recombinant proteins by heterologous expression in *Escherichia coli* [71]. However, as the requisite directed evolution experiments involved prolonged incubations in the presence of the amino acid [72], there were concerns that oxidation and reaction with cellular metabolites could complicate the selection procedure, and the similarity between δ-thiolysine and lysine may prevent selective recognition. To address these points the synthetase was initially evolved against a stable analogue with an acid-cleavable protecting group bonded to the Nε amino group. This served as a recognition element to ensure selective incorporation by an evolved *Mb*PylRS/tRNA_CUA_ pair.

By a combination of directed evolution and rational design, a mutant *Mb*PylRS/tRNA_CUA_ pair was identified that efficiently incorporated a designed precursor to δ-thiolysine. Upon cellular lysis the incorporated precursor fragmented and liberated the desired 1,2-amino thiol moiety that could undergo EPL with Ub1-76-SR [70] (Figure 2C).

Using the above procedure, δ-thiolysine was incorporated into Ub in place of K6 and in the Ubl SUMO2 at position K11. EPL was efficiently carried out with Ub1-76-SR prepared by intein technology and the δ-thiol was chemically removed post ligation by free-radical desulfurization [69]. These procedures furnished K6-linked diUb and SUMO2 ubiquitinated at position K11 (Ub-^11^SUMO2), both entirely of recombinant origin. Circular dichroism and biochemical assays were used to validate the physiological integrity of the product [70].

The Ub-^11^SUMO2 conjugate prepared by the genetically directed traceless approach subsequently found utility in revealing unexpected isopeptidase activity of Ub C-terminal hydrolase (UCH) family DUBs [52]. Arsenic-induced formation of Ub-^11^SUMO2 on the promyelocytic leukaemia protein (PML) leads to resolution of acute promyelocytic leukaemia (APL) [14]. The physiological DUB that reverses this conjugation is unknown. A study was carried out to identify DUBs that cleave Ub linearly fused to the N-terminus of proteins, a product generated by the action of the E2 conjugating enzyme UBE2W [73–75]. Surprisingly, UCH family DUBs were found to have peptidase activity towards linear fusions of Ub with UBE2W and linearly fused tetra-SUMO chains (Ub-SUMOx4) [76]. This was unexpected because UCH DUBs are inactive towards polyUb and it had been proposed that they only cleave small peptide remnants linked to the Ub C-terminus [77–80]. Importantly, UCH DUBs have been strongly associated with cancer and neurodegeneration but their substrate scope is poorly defined. Steady state kinetic analyses revealed that the specificity constant of UCH-L3 towards Ub-SUMOx4 was 5.76×10^3^ M^−1^·s^−1^ [73]. Ub-^11^SUMO2 prepared by the genetically directed method was tested as a substrate of UCH-L3 and was also cleaved by UCH-L3 with a specificity constant of 2.08×10^3^ M^−1^·s^−1^. This study revealed that UCH DUBs not only have the capacity to cleave Ub from the N-terminus of intact proteins, but can also cleave Ub isopeptide-linked to intact proteins. This highlighted the importance of utilizing a diverse array of ubiquitinated substrates when characterizing DUB activities and the readily available eight Ub linkages cannot always serve as surrogate substrates to provide conclusive readouts of DUB activity. The genetically directed method for traceless ubiquitination of proteins will be valuable for broadening the toolkit of model ubiquitinated substrate proteins and thereby accelerate our understanding of DUB activity determinants.

Limitations with the genetically directed traceless technology are that a bespoke amino acid is involved that requires a multistep synthesis [70]. Furthermore, some sites in proteins may not be compatible with incorporation of the sterically encumbered amino acid precursor and some sites are simply not amenable to amber suppression in general. However, efforts in our lab have streamlined the synthetic procedure and reengineered *E. coli* strains should improve incorporation issues [81].

A drawback with NCL/EPL in general are the inherently slow kinetics of the reaction [82,83]. Reactant concentrations should approach millimolar concentrations and the reactive groups should be well exposed. For folded substrate proteins that have poor solubility, efficient conjugation could thus be challenging. However, strategies exist that could be used to prepare Ub that is activated even more so than the thioester typically employed in EPL. For example, selenoesters undergo NCL/EPL with 1,2-amino thiol moieties orders of magnitude faster than thioesters [84]. The use of Ub selenoesters could negate the reduced reaction rates expected with folded proteins at low concentrations. Also, to produce an entirely native linkage, a desulfurization step is required. Therefore, Cys residues present in the substrate could also be desulfurized to alanine and that might have undesirable consequences. However, desulfurization may not always be required as despite the presence of a thiol group on the  δ C-atom of the modified lysine residue, the technology produces a native isopeptide linkage that is still cleavable by DUBs [85].

### Native isopeptide conjugates by Staudinger ligation

**Figure 3:**
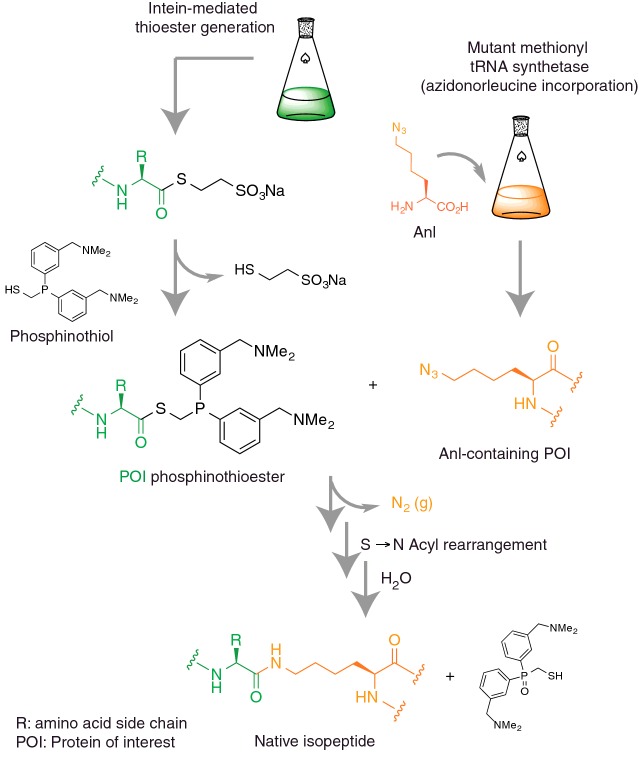
Traceless Staudinger ligation for isopeptide bond formation Genetic incorporation of a lysine derivative with Nε azido functionality (Anl) into recombinant protein and synthesis of a Ub C-terminal phosphothioester (via intein technology) generates the mutually reactive functional ligation handles. Staudinger ligation involves the liberation of nitrogen, an S to N acyl shift and hydrolytic oxidation of phosphorus resulting in the formation of a native isopeptide bond.

An elegant approach recently described in a methods paper from the Raines laboratory that should complement the above procedure also allows formation of a native isopeptide between recombinant proteins [86]. The traceless Staudinder ligation is a bioorthogonal amide bond-forming reaction between an azide and a phosphinothioester [87,88]. The extension involves the incorporation of a lysine surrogate that carries an Nε azido group (azidonorleucine, Anl) that can be incorporated into recombinant substrate proteins in bacteria in response to an ATG codon using a mutant methionyl-tRNA synthetase [89] (Figure 3). In parallel, Ub carrying a C-terminal phosphinothioester group is prepared by intein technology. The complementarity of the azide and the phosphinothioester permits traceless Staudinger ligation thereby forming an isopeptide bond. Staudinger ligation is compatible with aqueous buffer systems and near neutral pH so, in principle, conjugation could also be carried out under entirely native conditions on folded proteins.

However, the article is merely a conceptual protocol so its utility remains unknown although the Staudinger ligation has successfully been used for the linear semisynthesis of proteins [90,91]. The Staudinger ligation also suffers from relatively slow kinetics and as such may have limited utility [87]. Any additional Met residues must also be mutated to an alternative amino acid to prevent multiple coupling. Furthermore, the phosphinothiol and the azidonorleucine need to be synthesized as they are not readily available. It will be interesting to see what future this protocol has as it certainly has great potential.

### Genetically encoded orthogonal protection and activated ligation (GOPAL)

**Figure 4:**
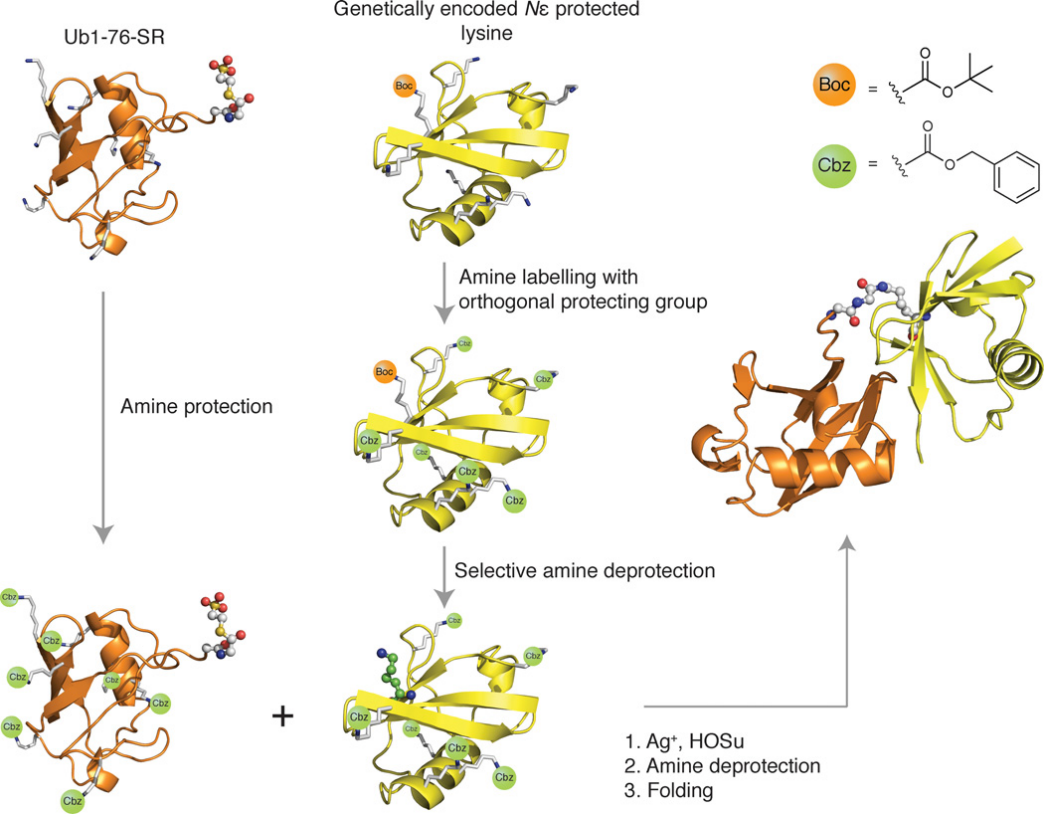
GOPAL strategy for site-specific isopeptide bond formation as exemplified for a diUb conjugate Site-specific genetic incorporation of an Nε *B*oc-protected lysine derivative into Ub (yellow) is followed by global orthogonal protection of the remaining amine functionality with Cbz-protecting groups, and subsequent acidic deprotection of the Boc-protecting group to generate a free lysine side chain at the genetically programmed site. Parallel production of Ub thioester, Ub1-76-SR (orange) is followed by global amine Cbz-protection of remaining amine functionality and subsequent silver-mediated aminolysis ligation with the selectively deprotected Ub species (orange) to generate the native isopeptide bond. The native conjugate is obtained after global deprotection of the remaining Cbz-protecting groups and protein refolding [*t*-butyloxycarbonyl (Boc), benzyloxycarbonyloxy (Cbz), *N*-(hydroxy)succinimide (HOSu)].

Another approach for genetically directing chemical ubiquitination has been described termed genetically encoded orthogonal protection and activated ligation (GOPAL) [92]. GOPAL uses a different principle to the methods described thus far. Rather than the installation of a lysine surrogate that has inherent chemoselectivity for a C-terminally activated Ub, selectivity towards a specific lysine Nε amino group is enforced using a chemical protection and selective deprotection regime (Figure 4). At the site of ubiquitination, the native *Mb*PylRS/tRNA_CUA_ pair system is used to direct the incorporation of lysine bearing an acid labile protecting group into Ub. All other instances of the amino group are then readily protected by chemical labelling with a protecting group that can be removed with conditions that are orthogonal to those required for removal of the genetically installed protecting group. This enables the selective deprotection at a genetically defined site yielding a Ub species with a single amine group. In parallel, C-terminally activated Ub1-76-SR is prepared and the same chemical labelling reaction is used to protect all instances of the amino group. The two components are then mixed and *in situ* silver mediated thioester conversion to a highly reactive, but amine specific, succinimidyl ester efficiently acylates the selectively deprotected Nε amino group forming an isopeptide bond [93]. The protecting groups installed by chemical labelling are then removed by incubation in an acidic cocktail [94]. The polypeptides are then isolated and folded and diUb can be purified by ion-exchange chromatography.

This technology enabled the production of atypical K6-linked and K29-linked diUb although it has subsequently been used to produce all atypical isopeptide linkages [80,92,95]. Access to K6-linked diUb enabled the determination of its crystal structure that was consistent with subsequent NMR studies using enzymatically prepared material [32]. The asymmetric nature of the diUb structure enabled iterative modelling of an extended K6-linked polymer. Intriguingly, this suggested that extended K6 polyUb chains might form helical filamentous structures. Access to atypical chains also enabled the first comprehensive specificity profiling of DUBs that had previously only been characterized for activity against K48- and K63-linked Ub. Indeed, the ovarian tumour (OTU) family DUB TRABID, implicated with Wnt signalling, was assumed to be a K63-linkage specific DUB [96]. Profiling against a more comprehensive panel of Ub linkages revealed that TRABID had significantly higher activity towards the K29 linkage. Kinetic analyses revealed that TRABID was in fact 40-times more active towards the K29 linkage relative to the K63 linkage (*k*_kcat_/*K*_m_=1.0×10^5^ M^−1^·s^−1^ compared with 2.5×10^3^ M^−1^·s^−1^), suggesting that the Wnt signalling pathway may be regulated by atypical K29-linked chains. Subsequent experiments revealed that TRABID also had high activity towards K33 linkages [97].

K29-linked diUb synthesized by GOPAL also provided intriguing insight into the polyUb linkage preference of an OTU deubiquitinase (vOTU) encoded by Crimean Congo haemorrhagic fever virus (CCHF) [98]. It was found that vOTU had activity towards K6, K11, K48 and K63 linkages but was inactive towards K29 and Met1 linkages. This raised the possibilities that K29 and Met1 linkages do not play a role in the antiviral response and/or they may in fact facilitate CCHF replication in infected host cells.

The GOPAL technology has been adopted by other laboratories and has subsequently undergone evolution [80,95,98–102]. The protecting groups installed can now be removed by mild catalytic methods with improved efficiency [103]. GOPAL also allows the preparation of tetrameric and branched Ub chains and also allows monomer-specific modifications to be made [95,99]. For example, it was demonstrated that any of the Ub monomers in a K11-linked chain could be isotopically labelled in a selective fashion to provide defined signals when carrying out structural characterization by NMR [103]. This should prove to be particularly valuable for structurally characterizing the recognition of polyUb chains by DUBs and Ub receptors.

Recently, the refined GOPAL methodology enabled further analysis of structure and dynamics of non-canonical Ub linkage types in solution [104]. The generation of isotopically labelled Ub dimers enabled generation of population-weighted conformational ensembles from NMR and small angle neutron scattering (SANS) data. Though characteristic ensembles were found for each chain type, a significant degree of overlap was found between the atypical and K48 or K63 structural ensembles. In short, similar Ub-dimer arrangements were also found between K6- and K11-linked diUb (K6Ub_2_ and K11Ub_2_) and between K29- and K33-linked diUb (K29Ub_2_ and K33Ub_2_). Several conformations of K6Ub_2_, K11Ub_2_ and K27Ub_2_ resembled K48Ub_2_ bound to the Ub receptor UBA2 and conformations of K27Ub_2_, K29Ub_2_ and K33Ub_2_ resembled K63Ub_2_. This led the authors to propose the idea that redundancy of function is inherent with polyUb signalling and suggests possible overlap in biological function [104].

### Production of heterologous chains by GOPAL

As GOPAL technology requires extensive chemical manipulation of the polypeptides, and the produced conjugate must be compatible with refolding, there were uncertainties over its utility beyond Ub chains. Excitingly, it was demonstrated that it could be extended to heterologous Ub-Rub1 chains [100]. Rub1 is the yeast orthologue of human NEDD8, a Ubl that regulates Cullin E3 ligase activity and that may also have cullin-independent functions [105,106]. Despite the occurrence of rubylation of Ub and ubiquitination of Rub1 under cellular stress conditions [12,13], the physiological significance of these modifications and the potential cross-talk between rubylation and ubiquitination is not well understood. To begin to generate tools to address questions towards the biological relevance of Rub1 and Ub cross-talk, GOPAL was used to prepare heterologous Ub/Ubl dimers of Rub1 and Ub where Ub was rubylated at K29 (Rub1–^29^Ub) and K48 (Rub1–^48^Ub). Rub1 ubiquitinated at K48 was also prepared (Ub–^48^Rub1) [100].

Interaction of the known K48 Ub binding domain UBA2 (from the proteasomal shuttle protein hHR23a) with Rub1–^48^Ub and Ub–^48^Rub1, but not Rub1–^29^Ub, was determined by NMR titration assays. Both heterodimers were shown to be structurally and functionally indistinguishable from the K48-linked heterodimer, the native Ub linkage to which UBA2 binds. The authors also showed that DUBs also had derubylase activity, as USP5 had high activity towards Rub1/Ub heterodimers. Other DUBs tested were more selective, exhibiting activity dependent on the position of the Ub moiety in either the proximal (OTUB1) or distal (UBP6 and USP2) position.

### Ubiquitination of non Ub/Ubl substrates with GOPAL

It has also recently been demonstrated that GOPAL can be used to site-specifically ubiquitinate proteins other than Ub/Ubls [101]. Dishevelled (Dvl) relays Wnt signals from the plasma membrane to various cytosolic effectors and the signalling activity of Dvl is governed by its DIX domain [107]. Wnt signalling regulates animal development and tissue homoeostasis and its dysregulation can result in cancer [108]. The DIX domain undergoes head to tail polymerization to assemble signalosomes which are responsible for relaying the Wnt signals [107]. Using the GOPAL methodology, recombinant, mono-ubiquitinated Dvl2 DIX was generated, with the Ub moieties site-specifically installed at two previously identified ubiquitination sites (K54 and K58) in human Dvl2 [109,110]. Correct folding was determined by circular dichroism measurements. This allowed the authors to establish that ubiquitination of the DIX domain at K54 inhibits Dvl oligomerization (though dimerization is possible) whereas oligomerization of the DIX domain ubiquitinated at K58 is unaffected. A crystal structure of the Dvl2 DIX domain was determined enabling these observations to be rationalized as UbK54 points into the DIX/DIX interface, where it was predicted to sterically impede the interacting DIX monomer, whereas K58 points away from this important interface.

Subsequent DUB profiling of the two respective ubiquitinated Dvl proteins discovered 28 DUBs (from all major DUB families) that could hydrolyse the DIX–Ub conjugates, with over half of those DUBs tested showing preference for Ub-^54^Dvl rather than Ub-^58^Dvl, including DUBs which only had previously demonstrated activity towards K11 or K63 polyUb chains. In contrast, Ub-^58^Dvl could only be hydrolysed by more indiscriminate DUBs and DUBs implicated with Wnt signalling. The results further highlight the deficiencies in using only Ub chains as tools for garnering meaningful insight into DUB activities.

GOPAL can now be used to prepare all Ub linkages and tetrameric K11-linked chains have been prepared. Recent developments, with the exception of K27-linked Ub, enable all Ub chains to be prepared by enzymatic reconstitution. However, there are still challenges associated with obtaining native chains of defined length. Although methods have been described for controlling the length of enzymatically assembled Ub chains [103,111], these methods are only compatible with assembly systems that produce a single linkage type with high fidelity. This is not the case with the current enzymatic systems for preparing K6, K11, K29 and K33 linked chains. Methods such as GOPAL should continue to be invaluable additions to the Ub biologists toolkit by enabling the production of heterotypic and heterologous chains, and otherwise inaccessible modified substrates, with the capability of introducing monomer-specific modifications with high precision.

## NON-NATIVE UBIQUITINATION

### Disulfide-directed ubiquitination and SUMOylation of histones

Protein engineering based on the humble disulfide bond has provided a platform for of an extremely powerful means to prepare Ub and Ubl conjugates. The heroic efforts involved in the described semisynthesis of uH2B restricted the generality of the procedure [61]. However, once the native conjugate was in hand it served as a reference standard to validate structural analogues prepared using simpler and more general approaches.

Parallel reports described an extremely versatile strategy for site-specifically ubiquitinating recombinant proteins under native conditions [112,113]. The caveats being that it produced a redox sensitive disulfide linkage that was slightly bulkier and longer than the native isopeptide (Figure 1 and Table 1). The substrate also could not contain additional cysteine residues as these would potentially undergo modification. To carry out this procedure the surrogate lysine residue was a cysteine residue introduced simply by mutagenesis. Full-length Ub thioester (Ub1-76-SR) was then prepared by intein technology. Ub1-76-SR was then subjected to an EPL-like reaction with the minimal 1,2-amino thiol species, cysteamine (Figure 2A). This procedure produced Ub bearing a thiol group that was amide-linked to its C-terminus (Ub-SH). Ub-SH could then be disulfide linked to the introduced Cys residue but to enhance the propensity of Ub-SH to undergo chemistry, it was ‘spring-loaded’ by the formation of a mixed disulfide with a low p*K*_a_ small molecule thiol_._ This was achieved by facile incubation of Ub-SH with bis(5-nitro-2-pyridyl) disulfide (DTNP) [112] or the related compound, bis(3-carboxy-4-nitrophenyl) disulfide (DTNB) [113], more commonly known as Ellman's reagent. Of note, DTNB offers improved aqueous solubility and, conveniently, disulfide formation can be monitored colorimetrically. Disulfide formation between activated Ub-SH and the cysteine-containing substrate was extremely rapid and compatible with folded proteins in physiological buffer at modest micromolar concentrations.

This method enabled the disulfide-directed ubiquitination of H2B at K120 (uH2B_SS_) and unlike the previous work, the procedure to produce uH2B_SS_ was expedient and could be carried out on intact recombinant histones under non-denaturing conditions, without the need for protracted semisynthetic techniques [112]. Histones were particularly amenable to this approach as only histone H3 (H3B) contains a single cysteine residue that can be mutated without consequence. Reconstituted nucleosomes containing uH2B_SS_ stimulated Dot1L methyltransferase activity towards H3K79 to a similar extent as nucleosomes containing native isopeptide-linked uH2B. This result demonstrated that the easily implementable disulfide-directed ubiquitination strategy could have broad utility for studying the effects of protein ubiquitination in general. Importantly, the facile and general nature of the approach enabled the production of a panel of H2B species Ub-modified at various lysine positions proximal to K120 (K108, K116, K125), including a K22 site on a histone 2A (H2A). Strikingly, modification of K125 on H2B and K22 on H2A both stimulated Dot1L methyltransferase activity to a similar extent (∼85%) as uH2B_SS_.

The conjugate uH2B_SS_ was also used to monitor the effects of H2B ubiquitination on higher-order chromatin compaction [114]. Extended nucleosomal arrays, representative of chromatin fibres, were prepared containing uH2B_SS_ or unmodified H2B and effects on compaction were determined by sedimentation velocity experiments. The presence of uH2B_SS_ was found to significantly decrease the degree of chromatin compaction to a similar extent to that observed with histone 4 (H4) acetylation [115]. Biochemical accessibility of the nucleosomal array was also determined by measuring Dot1L mediated H3K79 methylation and it was found that ubiquitination of H2B also stimulated methylation in the context of a chromatin fibre.

To obtain higher resolution information about chromatin fibre conformation, a homo-FRET assay was established that enabled monitoring of fluorescence emission steady-state anisotropy (SSA) of fluorescently labelled nucleosomal arrays. Decrease in the monitored SSA signal signified a decrease in internucleosomal distances and as such, chromatin fibre compaction. Nucleosomal arrays containing fluorescently labelled uH2Bss, hyperacetylated H4 (AcH4) or both modifications, were used to study the effects and potential interplay between the two histone PTMs. In comparison with unmodified H2B, distinct profiles were obtained for uH2Bss and AcH4, both of which demonstrated reduced levels of compaction. This appears to relate to potentially different mechanisms of array compaction elicited by the distinct PTMs. The effects of chromatin compaction upon nucleosomal arrays containing uH2B_ss_ were shown to be Ub-specific rather than a result of generic steric bulk effects. The disulfide connectivity of the Ub modification enabled facile redox-induced ‘deubiquitination’ of uH2Bss resulting in SSA profiles corresponding to fully compacted nucleosomal arrays. The SSA profiles for mixed uH2Bss/AcH4 modified nucleosomal arrays were indistinguishable from AcH4-only. In this case, deubiquitination did not lead to an SSA profile indicative of fibre compaction but remained unaltered, indicating that the PTM effects upon chromatin fibre compaction are non-additive and that AcH4 is dominant over uH2Bss.

Recent follow up studies explored the significance of distinct patches on the Ub surface by carrying out alanine scanning [116]. This study required a high-throughput approach for the preparation of site-specifically ubiquitinated nucleosomes. To address this, the protocol was streamlined such that nucleosomes were reconstituted first with cysteine mutant histone and were subsequently modified with ‘spring-loaded’ Ub-SH in their intact form. The stoichiometry of modification was compromised with this approach but was relatively consistent resulting in acceptably efficient (50–70%) modification. Importantly, control experiments revealed that the presence of unreacted cysteine mutant histone, ‘spring-loaded’ Ub-SH or the small molecule thiol byproduct of the disulfide-directed ubiquitination reaction, did not interfere with Dot1L methyltransferase activity. This provided an efficient way to screen intact nucleosomes containing 13 Ub patch mutations for their ability to stimulate Dot1L-mediated methylation of K3K79.

Contrary to the archetypal role of the Ile44/Leu8 patch in Ub on mediating biochemical processes, alanine mutagenesis at this position resulted in comparable levels of Dot1L activity to control sample [116]. The study mapped the C-terminal region of Ub consisting of residues 71–74 (LRLR) as being important for activity with L71 and L73 being the critical determinants. A second region consisting of residues 37–39 (PPD) also demonstrated reduced activity. Interestingly, these two patches were in close spatial proximity indicating that methyltransferase activity on chromatin is dependent on a functional hotspot at the Ub C-terminus. A similar requirement for methyltransferase activity was observed with the methyltransferase Set1 that methylates H3 at position K4, also in an uH2B dependent manner [117]. Interestingly, nucleosomes modified with the LRLR alanine mutant Ub underwent uH2B-dependent chromatin fibre compaction similar to wild type Ub. This illustrated that Ub molecule mediates chromatin regulation through multifunctionality.

Subsequently, other research labs have pursued the disulfide-directed strategy to ubiquitinate other histone proteins. As an example, a simplification of the disulfide strategy that simply uses a Ub G76C mutation (that further increases structural perturbation) was used to gain rapid access to ubiquitinated histone 2A (H2A) [118] which allowed investigation and corroboration of complementary experiments into the role of H2A ubiquitination in recruitment of 53BP1 to DNA double-strand-break (DSB) sites located near chromatin.

The extension of the disulfide-directed strategy to other Ubl-proteins has also been demonstrated. Through the use of similar chromatin compaction and oligomerization experiments described earlier [114], the generation of homogenous, site-specifically SUMOylated histone 4 at Lys12 (SUMOH4K12_SS_) provided the first insights into the structural effects of H4 SUMOylation upon incorporation into nucleosomal arrays. Namely that SUMOylation at this position inhibits nucleosome array folding and oligomerization. The study revealed that the mechanism by which SUMO modification stimulates inhibitory activity upon chromatin compaction is different from the mechanism of chromatin compaction displayed by H4 acetylation [119].

### Disulfide-directed ubiquitination of PCNA

A parallel study based on the first disulfide-directed strategy further highlighted its generality and utility [113]. During DNA replication minor lesions that have failed to be repaired need to be tolerated to prevent unnecessary cell death. Proliferating cell nuclear antigen (PCNA) is a trimeric ring-shaped protein complex that encircles the DNA duplex and overlooks the status of the replication fork [120]. Genetic studies had suggested that when a bulky DNA lesion is encountered, translesion synthesis (TLS) is initiated by mono-ubiquitination of PCNA at K164. This results in the recruitment of the TLS polymerase η (Pol η) that has increased substrate tolerance at the expense of fidelity of DNA replication. An error-free mechanism known as template switching can also occur and this is believed to be triggered by K63-linked polyubiquitination of K164 by an unknown mechanism [120]. PCNA can also be modified with SUMO at K127 and K164 which prevents recombination during S-phase [121,122].

At the time, natively ubiquitinated PCNA could be carried out by *in vitro* enzyme reconstitution but was extremely inefficient thus preventing biochemical experimentation [113]. The initial study showed that ubiquitinated PCNA could be readily prepared in large quantities. As the disulfide-directed ubiquitination reaction takes place on native protein, one can be confident that protein folding is not compromised. *In vitro* polymerase exchange assays revealed that disulfide Ub modified PCNA at K164 (Ub-^164^PCNA_SS_) recruited Pol η similarly to wild type Ub-PCNA, thereby highlighting the validity of the disulfide strategy in this context. Similarly to the initial study of uH2B, attachment of the Ub to multiple sites also mediated polymerase exchange illustrating plasticity in the mechanism of Pol η recruitment by Ub-PCNA. This also explained why highly perturbed analogues of ubiquitinated PCNA were biochemically functional [123]. Attachment of the Ubl SUMO at position K127 or K164 was found to have no effect on template switching thereby confirming that this was indeed a Ub-dependent process.

### Disulfide-directed polyubiquitination of PCNA

The versatility of the disulfide-directed approach also enabled the preparation and study of PCNA modified with K63-linked polyUb [124]. The strategy involved combining the disulfide-directed approach with chemoenzymatic production of K63-linked diubiquitin building blocks. This granted the ability to attach polyUb chains of defined length. Although K63-linked chains can be readily prepared by enzyme reconstitution of E1 activating enzyme and the K63-specific heterodimeric E2 complex UBE2N–UBE2V1 [21], a mixture of chain lengths are produced. Early work from the Pickart lab showed that enzymatic assembly of K63-linked (and K48-linked) polyUb chains can be controlled by supplying two Ub species where one is blocked at the C-terminus and one is blocked at K63 by cysteine mutation [111]. This enforces the production of a single product which in this case is a K63-linked diubiquitin molecule. The C-terminus can be readily ‘deblocked’ with a UCH family DUB, and the C63 site is ‘deblocked’ by its conversion to a pseudo lysine residue by alkylation with ethyleneimine [63,111]. In the PCNA study, the C-terminus of Ub was blocked by virtue of the thiol appendage present in Ub-SH. The C63 site in the second Ub was photocaged by alkylation with *p*-nitrobenzyl bromide producing a K63 diubiquitin that was linked via a native isopeptide and outfitted with a C-terminal thiol group (^PCK63^Ub-^63^Ub-SH). The cysteine could then be conveniently decaged by photo irradiation with low energy UV light allowing iterative extension of the K63 chain by additional rounds of disulfide-directed reaction with ‘spring-loaded’ Ub-SH or ‘spring loaded’ ^PCK63^Ub-^63^Ub-SH producing triUb or tetraUb modified PCNA, respectively.

*In vitro* assays were carried out allowing the role of polyUb chain length on DNA lesion tolerance to be systematically measured by observing Pol η read-through of an abasic site in a DNA template. As expected, mono Ub modification of PCNA promoted read-through whereas di-, tri- and tetra-modified PCNA demonstrated significantly, and progressively, reduced read-through of the DNA lesion. However, affinity of PCNA towards Pol η increased as the chains became longer. As only a single Ub binding domain is present in Pol η, enhanced affinity was assumed to be a consequence of avidity effects. As read-through was reduced with longer chain lengths it was proposed that longer chains sequester and trap Pol η in a non-productive configuration thereby inhibiting TLS. Strikingly, polymerase switching assays revealed that extended K63 chains reduced the capacity to switch from Pol δ to the error-prone TLS polymerase Pol η. These results provided novel insight into how polyubiquitination of PCNA at K164 promotes an error-free mode of replication.

### Disulfide-directed ubiquitination of  α-synuclein

Another example of the utility of the disulfide-directed approach was a study of multiple ubiquitinated forms of the intrinsically disordered protein α-synuclein (αSyn) [125]. The presence of protein aggregates in neurons, known as Lewy Bodies, rich in αSyn are the hallmark of a number of neurodegenerative conditions termed synucleinopathies [126]. These conditions include Parkinson's disease (PD) and dementia with Lewy bodies (DLB). Lewy bodies also contain ubiquitinated forms of αSyn and multiple ubiquitination sites have been identified. However, whether ubiquitination at distinct sites contributed to pathogenicity was unknown. The conjugate αSyn ubiquitinated at K6 had been prepared previously by a challenging three component semisynthetic procedure requiring chemical peptide synthesis [127]. This study revealed that ubiquitination at K6 of αSyn stabilizes the monomeric form of the protein and thus prevents its oligomerization and fibrillogenesis *in vitro*. However, the facile nature of the disulfide-directed ubiquitination strategy allowed a more comprehensive study as all nine of the known modified forms could be readily prepared and used to establish whether ubiquitination at a particular site, or sites, gave rise to fibrillogenesis and hence pathogenicity [125]. A potentially costly caveat with this approach however, is that without natively linked reference samples, erroneous conclusions can be drawn due to the non-native linkage behaving unexpectedly. Nevertheless, all nine identified ubiquitination sites were mutated to cysteine (K6C, K10C, K12C, K21C, K23C, K32C, K34C, K46C and K96C) in recombinant αSyn and were then ubiquitinated by reaction with activated Ub-SH. The conjugates could be prepared in milligram quantities.

Propensity for each of the ubiquitinated forms to aggregate was assayed by CD, thioflavin T (ThT) fluorescence and TEM. Results from the three independent assays were largely consistent with one another and revealed that protein modified at K10C and K23C readily formed fibrils and behaved like unmodified αSyn. However, modification at C6, C12 and C21 could inhibit fibril formation, and modification at C32, C34, C43 and C96 strongly inhibited fibril formation. These data suggested that modification of αSyn within regions that make up the core of the fibril fibre (residues 22–36 to 90–98) [128–130], prevent fibrillogenesis, whereas modification sites near the N-terminus (K6, K10 and K12) do not. Additionally, ubiquitination sites near fibril boundaries have fibre forming properties (e.g. K23) or can promote oligomerization (K96). It was suggested that the inhibitory effect of these modifications could be a result of Ub sterically interfering with significant aggregation intermediates such as long-range interactions with the N-terminus that are proximal to the ubiquitination sites. Masking of N-terminal lysine charges that may be involved in interactions with the highly negatively charged C-terminus or ions in solution were also proposed as potential inhibitory mechanisms.

Follow up studies by the same lab using the disulfide-directed strategy determined the contribution of the same nine ubiquitination sites on αSyn towards propensity for proteasomal turnover [131]. The authors concluded that site-specific modification of αSyn with Ub supports varied levels of αSyn degradation with N-terminal modifications, K12, K6, K21 leading to the most pronounced levels of degradation compared with other positions investigated.

More recently, SUMOylated αSyn was prepared using both SUMO1 and SUMO3 [132]. In cell culture, αSyn SUMOylation has been mapped to K96 and K102 and Lewy bodies are immunoreactive to SUMO1 [133–135]. SUMOylation at K102 of αSyn inhibited aggregation more significantly than SUMOylation at K96 and modification with SUMO1 was more inhibitory than SUMO3. The identification of isoform-dependent and SUMO site-specific effects upon αSyn aggregation are in contrast with ubiquitination of αSyn, where K102 is not a physiological site [125]. This study has now identified SUMO1ylation at K102 as an attractive target for therapeutic intervention towards tackling synucleinopathies [132].

Interestingly, the recent use of the disulfide-directed strategy to generate Ub dimers allowed a kinetic analysis of enzyme-independent ubiquitination and demonstrated a correlation with linkage abundance in yeast [136]. This suggested that intrinsically accessible lysines within Ub were selected for prevalent cellular functions. Disulfide methods have also been use to show that site-selective mono-ubiqutination of the GTPase, Ras decreases protein sensitivity to GTPase-activating protein (GAP)-mediated hydrolysis [137].

### Ubiquitin chains using thiolene chemistry

Another approach for preparing Ub conjugates uses thiolene chemistry [138,139]. This approach is conceptually similar to the disulfide-directed strategy with distinct advantages and disadvantages. In this approach, a cysteine residue is also introduced as the lysine surrogate but it is reacted with Ub bearing a terminal vinyl (ene) group. The latter can be readily prepared using intein technology. In short, the Ub is activated at the C-terminus as a thioester which can then undergo a simple aminolysis reaction with an excess of small molecule amine [140]. To append the ene functionality allylamine was employed [138]. The functionalized Ub can also be prepared using a complementary approach utilizing a UCH family DUB, via a transamidation reaction with allylamine [141]. Since 1905 it has been appreciated that substituted enes can undergo free-radical addition with thiols forming a thioether, a reaction known as thiol-ene chemistry [142]. Thiol-ene chemistry has seen a resurgence over the past years as it meets many of the requirements of desirable ‘click’ reactions (i.e. processes that work under operationally simple, oxygen- and water-tolerant conditions, and generate products in high yields with minimal requirements for product purification), but mainly in the area of polymer chemistry. Importantly, the reaction produces the anti-Markovnikov product (i.e. the thiol sulfur adds to the terminal C atom of the ene forming a linear rather than a branched linkage) that gives a good impression of the native lysine side chain. However, like the disulfide linkage, it is slightly longer than the native isopeptide but less bulky (Figure 1). A significant advantage over the disulfide linkage is that it is redox stable. However, it has not been demonstrated that thiol-ene coupling can be used to ubiquitinate proteins other than Ub itself and as such has only been used to prepare Ub chains.

Recent biophysical analysis of thiol-ene produced Ub chains compared with native conjugates was carried out in order to ascertain the extent to which thiol-ene-generated Ub conjugates successfully mimic the native isopeptide bond. For the most part, high similarity between native and the non-native surrogates was found (by SAXS analysis) but DUB activity assays indicated that for OTUB1, the non-native surrogate was an unsuitable mimic of the native linkage [143].

## NON-HYDROLYSABLE Ub CONJUGATES

The archetypal click reaction involving the formation of a triazole linkage between azides and alkynes, known as copper catalysed azide-alkyne cycloaddition (CuAAC), has found huge utility in protein research [144,145]. Furthermore, numerous methods for incorporating the requisite azide and alkyne reactive handles into recombinant proteins have been developed [146–148], and these methods have been adopted in innovative ways to prepare Ub conjugates that cannot be hydrolysed by DUBs.

The first example where CuAAC was utilized for preparing a Ub/Ubl conjugate was between alkyne-functionalized SUMO2 prepared by semisynthetic methods and a synthetic peptide corresponding to a region known to contain a SUMOylation site in PML [149]. Subsequent work used a different strategy that enabled incorporation of both the alkyne and azide functionality at the genetic level allowing the production of recombinantly derived diubiquitins conjugated at all seven linkage sites [150] (Figure 1). Azide functionality was introduced adjacent to the C-terminus of a distal Ub using selective pressure incorporation [148]. The proximal Ub was prepared by amber codon suppression using the native *Mb*PylRS/tRNA_CUA_ pair that granted the incorporation of the alkyne-functionalized unnatural amino acid, propargyloxycarbonyl-L-lysine (ProcK) [146]. Folded Ub molecules were purified and coupled by CuAAC. Follow up studies demonstrated the ability to ubiquitinate substrate proteins and was exemplified by the modification of PCNA at K164 and polymerase β (Pol β) at position 61 [151,152].

Evolution of this technology allowed its deployment for the production of polymeric Ub chains [45] and detailed experimental procedures have been reported [153]. The evolved procedure involves incorporation of azide and alkyne functionality into the same Ub molecule by carrying out amber codon suppression and selective pressure incorporation simultaneously. This yielded 0.5–2.0 mg of bifunctional protein per litre of culture medium. CuAAC could then be carried out resulting in the production of non-hydrolysable Ub polymers linked at K11, K27, K29 or K48 positions [45]. Importantly, the K48 polymeric species was recognized by a K48-specific antibody whereas the K11, K27 and K29 linkages were not. Polymers linked at other sites were not prepared nor was a K11-specific antibody tested against the panel [154]. Excitingly though, it was demonstrated that in a one-pot reaction, a recombinant substrate protein containing ProcK could be site-specifically modified with non-hydrolysable polymeric Ub chains. The exemplar substrate in this case being Pol  β. This technique should have broad utility for assessing the role of Ub modification in protein function and assigning DUBs to substrates for example.

The authors exploited the non-hydrolysable nature of the linkages and used them as inhibitors of DUBs in *Xenopus laevis* extracts. The process under investigation was the degradation of the cell cycle regulator Cyclin B in response to Ca^2+^ activation of the multi-subunit anaphase promoting complex/cyclosome (APC/C) E3 ligase. It is known that Cyclin B is typically modified with K11-linked Ub chains that ensure its cell cycle-dependent degradation [155]. *Xenopus* extracts were treated with K11, K27 and K29 non-hydrolysable chains prior to APC/C activation. Interestingly, K11-linked polymers potently inhibited Cyclin B degradation, presumably by binding to the proteasome and inhibiting its activity, whereas K27- and K29-linked polymers did not. It was proposed that K27- and K29-linked chains do not serve as proteasome targeting signals as they would also be expected to inhibit Cyclin B degradation if this was the case. Assessment of DNA morphology and spindle formation indicated that the meiotic state of the cell extracts had been perturbed by the addition of the non-hydrolysable K11 chains as evident from extension of the meiotic state owing to the inability to degrade Cyclin B. In comparison, buffer-treated extracts displayed typical interphasic nuclear morphology confirming exit from meiosis associated with Cyclin B degradation.

An alternative to this approach also involves amber codon suppression but with a different azide-functionalized unnatural amino acid [156,157]. Mutant substrate protein was prepared bearing azidophenylalanine in place of the acceptor lysine residue using an evolved *Methanocoldococcus jaanaschii* tyrosyl-tRNA synthetase/tRNA_CUA_ pair [147] (Figure 1). Complementary alkyne functionality was chemically appended to the C-terminus of Ub or a Ubl using the semisynthetic aminolysis strategy mentioned above but with propargylamine in place of allylamine. Using this approach the effect of SUMO2 automodification of the SUMO E2 conjugating enzyme UBE2I (also known as Ubc9) was studied. UBE2I is the cognate E2 partner for the SUMO E1 activating enzyme SAE (SAE1–SAE2). It was reported that autoSUMOylation of UBE2I on K14 with SUMO1 modulated its substrate specificity and attenuated its activity towards its E3-independent substrate RanGAP1 but increased it for a different substrate, Sp100 [158]. However, it was not known whether the SUMO2 isoform, that has 44% sequence identity with SUMO1, could also be regulated in this manner. The triazole approach was used to prepare homogenously SUMO2 modified UBE2I that was then used in direct biochemical experiments. Indeed, it was found that SUMO2 conjugated to UBE2I via a triazole linkage had reduced activity towards RanGAP and activity towards Sp100 was increased [156].

### Caveat emptor

As with all methods that produce a non-hydrolysable linkage, caution must be exercised when interpreting biological data using these tools. It is known that certain DUBs make intricate contacts with the linkage [37,159]. Furthermore, Ub recognition modules termed Ub-binding domains can exist as a series of repeating Ub-binding units which can only be complemented when a distinct Ub linkage is presented, as the repeating nature of the binding domain serves as a molecular ‘ruler’, and avidity-driven binding is only demonstrated when the correct linkage is present [160]. Any perturbation to the isopeptide linkage could disrupt this finely tuned mode of recognition such that the conjugate no longer measures up. Quantum mechanical and molecular mechanical models of native K48-linked diubiquitin and the triazole-linked analogue have revealed that the structures are largely similar although the triazole-linked conjugate has reduced flexibility [161]. Whether other linkage types can be acceptably recapitulated via triazole linkages remains to be determined. Comparative biophysical and structural analyses of native and triazole-linked conjugates would also help determine the utility of these chain types for exploratory biological experiments.

## ENZYME-INDEPENDENT UBIQUITINATION IN CELLULAR SYSTEMS

*In vitro* methods for ubiquitinating proteins offer precise control over the substrate, the site, the extent and the topology of the modification. Being able to afford this level of precision in the context of live cells, with spatiotemporal control, would enable exciting new possibilities for cell biology experimentation and address limitations with existing strategies. For example, E3s responsible for substrate modification are often unknown and even when they are known, the constitutive nature of gene disruption and the low temporal resolution of RNA knockdown and complementation approaches can result in lethality or adaptive responses that alter the (patho)physiology under investigation [162,163]. Novel experimental possibilities granted by the ability to trigger ubiquitination in cells with high temporal resolution would also enable the study of ubiquitination at various sites and their effect on substrate localization in real-time could be inferred. Furthermore, Ub regulated cell signalling networks could be dissected by obviating the requirement to activate upstream signalling components. The kinetics of ubiquitination-dependent downstream events could also be quantified.

**Figure 5:**
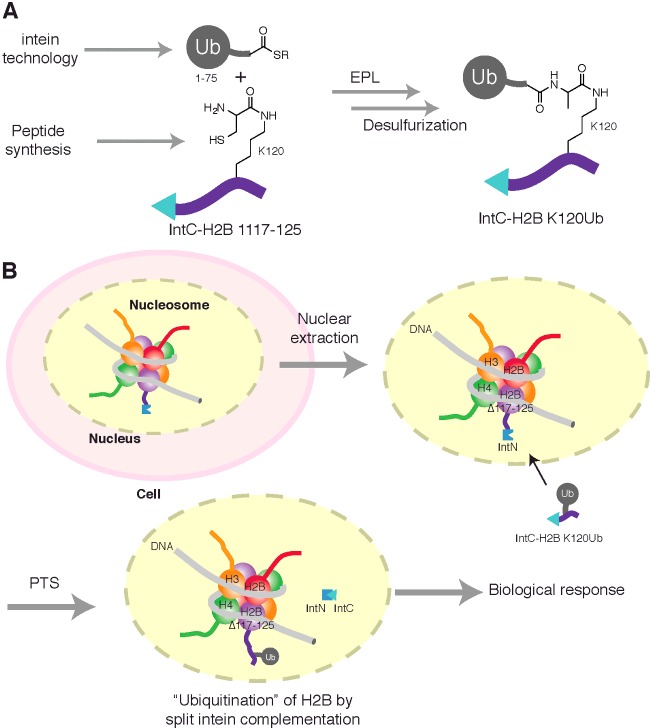
PTS methodology for installing Ub modifications in cellular systems The complementation of engineered IntN and IntC fusion proteins undergo splicing to generate a native peptide bond. (**A**) Generation of the exogenous IntC–ExtC fusion (IntC-ΔN_H2B-UbK120) harbouring an isopeptide-linked Ub was assembled by EPL between Ub1-75-SR and a lysine surrogate (at position K120) of the synthetic H2B 117-125 fragment. (**B**) The installation of a Ub modification upon H2B in cellular nucleosomes following nuclear extraction. Extracted nuclei containing exogenously expressed H2BΔC-IntN, that has been incorporated into chromatin, are permeable to protein cargoes. Delivery of, IntC-ΔN_H2B-UbK120 enables PTS to generate ubiquitinated H2B, eliciting a downstream biological response that can be measured.

Towards this goal protein trans-splicing (PTS) has been harnessed to install a Ub modification on H2B in cellular nucleosomes in purified nuclei (*in nucleo*) [164]. PTS is an extension of intein activity whereby the intein is split, either artificially or naturally, into N-terminal (IntN) and C-terminal (IntC) fragments that form a functional intein upon complementation [47]. Inteins are analogous to RNA introns as they catalyse their own excision from a polypeptide sequence with concomitant ligation of the N-terminal polypeptide (ExtN) with the C-terminal polypeptide (ExtC) via a native peptide bond. In the example with H2B, the ExtN fragment corresponded to the N-terminal bulk of the H2B protein but was devoid of the C-terminal nine residue tail that harbours the K120 ubiquitination site (H2BdeltaC) (Figure 5). This was exogenously expressed in HEK293T cells as an IntN fusion (H2BΔC-IntN). Various split intein systems were explored and that from the cyanobacterium *Anabaena variabilis* (Ava) gave the highest levels of H2BΔC-IntN fusion protein (∼10% total H2B) and the majority was found to localize to chromatin. The ExtC fragment corresponded to the nine residue C-terminal H2B tail with a Ub pre-attached at K120 via an isopeptide bond. This was fused to the C-terminus of IntC (IntC-ΔN_H2B-UbK120) (Figure 5). A lysine surrogate was incorporated at position K120 of the synthetic peptide that enabled EPL with Ub1-75-SR. Post-ligation, desulfurization of the thiol auxiliary introduced a C-terminal Ub G76A mutation, as discussed earlier, that also rendered the isopeptide more resistant to DUB isopeptidase activity.

In a proof of concept experiment, intact nuclei from cells expressing H2BΔC-IntN were isolated [164]. As the nuclear membrane is permeable to protein cargoes, the IntC-ΔN_H2B-UbK120 species could be delivered to the nucleosomes. Western blotting confirmed production of semisynthetic uH2B by the PTS mechanism. To confirm if the semisynthetic uH2B was functional, methylation levels of K3K79 were probed by immunoblotting. A 2-fold increase in H3K79 methylation was observed when all PTS components were present confirming *in nucleo*, enzyme-independent production of biochemically functional uH2B.

### Other methods for protein ubiquitination

Although this review has endeavoured to provide an informative overview on recombinant protein-based methods for protein ubiquitination that have garnered significant biological insight, there exists in the literature other examples of structurally divergent non-native isopeptide conjugates. Though having not formed a significant part of this review, certain examples are worth highlighting for their potential in generating insight into the Ub system, historic or otherwise. For example, the use of chemical methods that exploit the nucleophilicity of thiol functional groups present in cysteine amino acid side chains have been widely used to generate non-hydrolysable Ub conjugates of entirely recombinant origin. Cross-linking agents such as dichloroacetone [53,165–167] or Michael acceptors based on dibromo-maleimides and dibromo-pyridazinediones [168] have furnished Ub dimers of varied linkage identity (Table 1). Methods that enable formation of non-hydrolysable linkages with synthetic peptides have also been reported [169,170]. In many cases, the experimental utility of such conjugates has been largely superseded by EPL and GOPAL methods to make native isopeptide bonds, however, the non-hydrolysable nature of specific isopeptide mimetics make them attractive and powerful tools for investigating DUB activity and biology upon Ub chains or ubiquitinated substrates. Also, not an area highlighted herein but recently reviewed elsewhere [171], the generation of diUb conjugates as activity-based probes for profiling the specificity and activity of DUBs has been reported [172–176].

## SUMMARY

There are now several divergent approaches for chemically ubiquitinating substrates via a native isopeptide bond that pave the way for a new line of investigation into this fundamental post-translational modification. Methods reliant on synthetic peptide synthesis and those using recombinant technologies, complement one another as no single strategy can satisfactorily address all protein conjugates under investigation. Strategies for preparing non-hydrolysable conjugates allow distinct and insightful experiments to be carried out that cannot be achieved with their native counterparts. However, these conjugates should have high structural similarity with the native isopeptide bond such that they engage precisely the same biochemical processes.

The field has unquestionably reached a level of maturity whereby greater biological understanding of the Ub system can be obtained through use of the technologies described herein. Significant contributions have been made that provide great opportunity for those actively engaged in Ub research to carry out more direct and insightful experiments. However, despite the obvious progress there is no single strategy that can address all of the necessary requirements for rigorous and conclusive interrogation of the Ub system. Where appropriate and possible, the use of native conjugates should be utilized in order to ensure robust and conclusive data and that interpretation of experimental results are not obscured by deviation from non-physiological parameters.

Ub chains and ubiquitinated conjugates prepared via enzyme-independent methods are proven and established tools for studying ubiquitination. However, the functional relevance of ubiquitinated proteins produced by the methods described herein compared with those found *in vivo* is a valid query. For all of the examples described above that produced native isopeptide linkages, but required artificial protein folding, the structural integrity of the material was exhaustively validated by structural and biochemical analysis. Invariably, the examples that form an unnatural linkage are performed on natively folded material under non-denaturing conditions. Despite being confident that protein fold is not compromised in these latter cases, a potential caveat is that the isopeptide linkage displays compromised isostery with the native linkage. The significance and consequence of this methodological compromise is very dependent on the biological question being asked and should be considered on a case-by-case basis.

The next logical progression would be to extend the concept of enzyme-independent ubiquitination into live cells, thereby enabling the spatio and temporal aspects of ubiquitination to be studied with an unprecedented level of precision.

## Abbreviations

AcH4: hyperacetylated H4
Anl: azidonorleucine
APC/C: anaphase promoting complex/cyclosome
CCHF: Crimean–Congo haemorrhagic fever virus
CuAAC: copper catalysed azide-alkyne cycloaddition
DLB: dementia with Lewy bodies
DSB: double-strand-break
DTNB: bis(3-carboxy-4-nitrophenyl) disulphide
DUB: deubiquitinating enzyme
Dvl: Dishevelled
EPL: expressed protein ligation
ExtC: polypeptide C-terminal to intein
ExtN: polypeptide N-terminal to intein
GAP: GTPase-activating protein
GOPAL: genetically encoded orthogonal protection and activated ligation
H2A: histone 2A
H2B: histone 2B
H3: histone 3
IntC: C-terminal intein fragment
IntN: N-terminal intein fragment
NCL: native chemical ligation
NEDD8: neural precursor cell expressed, developmentally down-regulated 8
OTU: ovarian tumour
PCNA: proliferating cell nuclear antigen
PD: Parkinson's disease
PML: promyelocytic leukaemia protein
POI: protein/peptide of interest
Pol β: polymerase β
Pol η: polymerase η
polyUb: polyubiquitin
ProcK: propargyloxycarbonyl-L-lysine
PTS: protein trans-splicing
SUMO: small ubiquitin-like modifier
SUMOH4K12_SS_: disulfide SUMOylated histone 4 at Lys12
αSyn: α-synuclein
ThT: thioflavin T
TLS: translesion synthesis
Ub: ubiquitin
UBA1: ubiquitin E1 activating enzyme
Ubl: ubiquitin-like protein
UCH: ubiquitin C-terminal hydrolase
SANS: small angle neutron scattering
SSA: steady-state anisotropy
